# Reevaluating chronic opioid monitoring during and after the COVID-19 pandemic

**DOI:** 10.2217/pmt-2020-0063

**Published:** 2020-09-18

**Authors:** Prashant N Rao, Rohan Jotwani, Jatin Joshi, Amitabh Gulati, Neel Mehta

**Affiliations:** ^1^Department of Anesthesiology, NewYork-Presbyterian/Weill Cornell Medical Center, NY 10065, USA; ^2^Department of Anesthesiology & Critical Care, Memorial Sloan Kettering Cancer Center, NY 10065, USA

**Keywords:** COVID-19, opioid misuse, opioid monitoring, telemedicine, urine drug monitoring

The coronavirus disease 2019 (COVID-19) global pandemic has demanded an unprecedented reallocation of clinical and public health resources in the USA. This rapid redistribution of medical resources has compromised significant public health challenges our society had faced prior to the COVID-19 pandemic, including chronic pain and the related opioid epidemic. Population studies have demonstrated that greater than one-fourth of US adults suffer from chronic pain, amounting to over 50 million US adults [1]. Management of chronic pain with the use of opioids requires routine screening protocols that are frequently employed in a typical in-person visit, including psychological and behavioral screening, as well as quantitative urine drug screening [2]. However, in the setting of the COVID-19 pandemic which mandated social distancing, physicians initiated and continued opioid management for patients through the use of telemedicine. Though many regions in the USA have begun reopening, or will likely do so in the near future, it remains crucial to continue to limit exposure risk and reevaluate the methods of chronic opioid monitoring to accommodate patients who are at high risk for either contracting or suffering increased morbidity from COVID-19, such as elderly or immunocompromised patients. Furthermore, in the event of a resurgence of COVID-19 restricting non-emergent office visits, it is prudent to develop infrastructure and protocols to seamlessly provide optimal care and opioid monitoring for patients.

Remote opioid management has been limited to assessing and monitoring patients through telemedicine. Opioids continue to harbor significant public health risks, such as opioid misuse, overdose and diversion, with approximately 32% of opioid overdose deaths resulting from prescription opioids [3,4]. Thus, significant multidisciplinary approaches have been implemented prior to the COVID-19 pandemic to improve and optimize the safety of chronic opioid therapy, including reducing opioid prescriptions, identifying patients at high risk for misuse with various psychological assessments, reviewing statewide prescription drug monitoring program data employing urine toxicology monitoring and continuously reassessing the need for chronic opioid maintenance [4–12]. These challenges are further exacerbated in this clinical environment, considering that providers are unable to physically examine and evaluate their patients. Given the emphasis on social distancing and the possibility of a resurgence of COVID-19, we aim to highlight the evidence and best practices to continue comprehensive and effective opioid management during remote patient interactions to maximize safety for patients and providers, with an emphasis on drug monitoring regimens, while remaining cognizant of the public health challenges at hand.

## Is telemedicine an appropriate alternative for opioid management?

With social distancing precautions in effect, there is decreased personal and physical interaction between chronic pain patients and their providers, complicating the routine assessments made with chronic opioid therapy. Telemedicine, however, presents a unique opportunity for continuity of care, while minimizing COVID-19 exposure risk. Its role in chronic opioid therapy has already been established in multiple studies, often used to bridge the provider gap in rural areas for the treatment of opioid use disorder [13–16]. One study reported that patients receiving opioid agonist therapy (OAT) evaluated via telemedicine were approximately 27% more likely to be retained in therapy at 1 year compared with patients evaluated with in-person visits [13]. This higher retention rate in patients undergoing treatment for opioid use disorder may be alluding to the social and logistical barriers patients may face while undergoing OAT that requires face-to-face interactions [13,14]. Furthermore, previous studies have demonstrated that telemedicine was non-inferior to in-person encounters for patients undergoing OAT, evidenced by no significant statistical differences in additional substance use or average time to abstinence [17,18]. Recent consensus guidelines by Shanthanna *et al.* and Cohen *et al.* both emphasize the use of telemedicine platforms to facilitate patient interactions for opioid therapy during social distancing restrictions in light of the COVID-19 pandemic [19,20].

However, there exist concerns that telemedicine visits inherently differ from in-person encounters and do not foster the same patient–physician relationships. For example, some limitations of telemedicine may include compromised patient confidentiality depending on the patient’s social situation and the provider’s difficulty in ascertaining a patient’s social cues and/or signs of intoxication via a virtual encounter. There may be inherent differences in the quality of interactions and assessments capable with video versus telephone-based platforms. Further, assessment via the physical exam may be limited as the provider may be unable to perform common maneuvers to aid in the differential diagnosis, or the patient’s pain may limit active range of motion. Certain patients, specifically the socioeconomically disadvantaged, the underserved, and the elderly, may not have either the access or the skills to the technology necessary for telemedicine, creating barriers to care for high-risk chronic pain populations. Given these barriers, our practice has been able to mitigate these operational concerns by dedicating staff to assist patients in establishing the means to communicate with providers via telemedicine. Finally, some healthcare systems may not have the infrastructure in place to facilitate this method of communication in a confidential manner, though there are ample low-cost resources available, and the recent modification of federal regulations have made this more feasible in a timely manner [21].

Though telemedicine has limitations, we recommend that efforts must still be made to employ this tool when feasible in order to balance the risks of exposure during in-person encounters with the benefits of appropriate assessment and monitoring during chronic opioid therapy. Even in light of nationwide reopening, physicians and patients must continue to employ precautions to limit COVID-19 exposure risk whenever safe and possible, especially for those at high-risk of morbidity from COVID-19, such as, but not limited to, the patient populations described in Table 1, adapted from the CDC.

**Table 1.** Examples of patients at high risk for morbidity from COVID-19.

Patient examples
Elderly patients
Lung disease, for example, asthma, chronic obstructive pulmonary disease, pulmonary fibrosis, smoker
Obesity, for example, BMI >30
Heart disease, for example, coronary artery disease, cardiomyopathies, heart failure
Diabetes, Type I and Type II
Immunocompromised state, for example, genetic immunodeficienies, cancer, HIV, cystic fibrosis, etc.

## Should drug monitoring tests be utilized in conjunction with telemedicine?

Prior to the COVID-19 pandemic, objective drug monitoring was a mainstay during chronic opioid therapy, specifically urine drug monitoring (UDM) [5]. However, with limitations on in-person, nonemergent office visits and the implementation of social distancing, further evaluation of drug monitoring protocols are warranted in this unprecedented era. For example, the previous practices of random, in-office urine or saliva testing, point of care testing and quantitative follow-up testing may harbor more risk than benefit to patients and healthcare providers in light of COVID-19, but may be necessary for patients at high-risk of opioid misuse. We discuss the evidence and guidelines regarding UDM prior to COVID-19, as well as society recommendations at the start of the pandemic and aim to discuss novel and unique ways in which drug monitoring can be maintained in the post-COVID-19 era.

Expert consensus guidelines have recommended that the need and frequency of testing should be determined by risk stratification, including patient history and physical examination, previous opioid use and evidence of addictive behaviors, validated screening assessment tools, prescription drug monitoring programs and previous UDM if available [11,22–24]. Ultimately, the provider must determine the frequency of UDM, with increased risk necessitating increased frequency of UDM [11,12]. Given the possibility of sample tampering, strategies to mitigate that risk include openly discussing the rationale for UDM with patients, observed collections or random collections [11]. Traditional UDM testing typically requires face-to-face encounters, possibly with multiple providers, in a clinical office. However, this arrangement unduly increases the risk of exposure to COVID-19 for both patients and medical staff for nonemergent laboratory testing.

Although medical societies and governmental regulatory agencies recommend the use of UDM, there is a lack of clinical evidence regarding its utility in risk reduction for opioid misuse [10–12]. Recent guidelines by the American Society of Addiction Medicine (MD, USA) denote high risk situations that may warrant in-person urine testing as patients with known or suspected diversion histories, patients who present to their opioid treatment program (OTP) with signs of intoxication, patients with overdose history and patients with significantly unstable opioid use disorder [25].

Based on a comprehensive evaluation of the aforementioned risk factors, screening assessment tools conducted via telemedicine and previous behaviors, we recommend that pain management physicians should risk-stratify patients undergoing chronic opioid therapy and proceed with monitoring regimens based on the assessed risk.

## How can UDM be adapted for telemedicine?

Incorporating UDM into a telemedicine practice requires an understanding of a number of variables including UDM technology, frequency of testing, patient risk and ultimately when is there sufficient enough reason to warrant a transition from remote to in-person testing. Again, the American Society of Addiction Medicine denotes high risk situations that may warrant in-person urine testing as patients with known or suspected diversion histories, patients who present to their OTP with signs of intoxication, patients with overdose history and patients with significantly unstable opioid use disorder [25].

Consensus guidelines regarding UDM established in 2018 by Argoff *et al.* recommend at least annual testing for low risk patients and at least biannual testing for moderate risk patients [11]. Thus, for this cohort, it may be reasonable to consider having urine drug testing deferred for specific patients if there is a reasonable expectation for a planned in-person visit within the recommended testing period or to employ remote monitoring options when possible. For example, virtually-observed oral fluid-based tests or breathalyzer tests can be conducted via telemedicine or unobserved urine tests with subsequent validity testing can be employed to rule out sample tampering [26]. Additionally, more frequent telehealth encounters may present a valuable compromise for close monitoring and trending of risk stratification, which may help identify low-risk patients who are now becoming higher risk in this unprecedented era. Though these options may not be suitable for every patient, the benefits of continued drug monitoring with limited in-person interaction may make these options preferable in lower risk patients.

In higher risk patients for whom testing is deemed promptly necessary, there exist multiple options for testing, including both the remote options discussed above, as well various ways to perform in-person monitoring. Whether initiating or maintaining opioid medications for high-risk cohorts, it may be reasonable to consider a combination of frequent remote UDM and less frequent in-person UDM, in order to ensure validity of testing and a thorough evaluation. Discrepancies or inconsistent results might result in leaning more toward an in-person regimen, whereas consistent appropriate test results might warrant moving toward remote monitoring.

Additionally, in-person monitoring can be accomplished through a number of forms that do not require a patient visiting a high-risk healthcare facility such as a pain management clinic or hospital. Either healthcare systems or third-party companies may present the option of witnessed at-home testing, where a witnessed urine or saliva sample may be collected at the patient’s residence by a qualified collector, who should have adequate personal protective equipment available prior to conducting testing via this method. Another alternative may include having the patient present directly to a laboratory testing facility for observed, in-person testing.

Regardless of the specific in-person modality, it is important to note that in-person clinic visits require a great deal of coordination and attention to detail on behalf of the healthcare providers in order to decrease risk for both patients and providers. For example, adequate personal protective equipment should be readily available and clinic flow should be adapted in order to adhere to strict social distancing guidelines, facilitated by minimal wait times and appropriate appointment spacing to decrease crowding of clinics. Only if these measures can be adopted and adhered to, may it then be justifiable to conduct thorough, in-person opioid monitoring. It is also important to note that evidence regarding the efficacy and noninferiority of the above discussed technologies compared with traditional UDM is limited, but the risks and benefits of traditional testing in the COVID-19 era needs to continuously be weighed. However, the compelling need for social distancing in the era of COVID-19 will hopefully drive technology and innovation for large-scale adaptation for these remote testing modalities.

Overall, UDM can be adapted for telemedicine. For low risk patients, we recommend utilizing remote monitoring with virtually-observed oral fluid-based tests or unobserved urine tests with subsequent validity testing in conjunction with frequent virtual follow-ups to rule out opioid or substance abuse. For high-risk patients, we recommend that providers must balance the risks of less comprehensive opioid monitoring with the exposure risk of COVID-19 and can employ both remote and in-person monitoring modalities. When in-person encounters are deemed necessary for a safe treatment plan, it is crucial to adapt the workflow of the clinic to accommodate social distancing.

## How can providers mitigate the risk of opioid abuse in high-risk patients?

There are a number of practical strategies that providers can employ when prescribing opioid medications remotely to decrease the risk of opioid abuse. If initiating or maintaining opioid treatment for high risk populations, shorter (1–2 weeks) courses of opioids are recommended in that they necessitate follow-up and monitoring. Further, prescribing lower risk opioid class medications (such as tramadol), nonopioid adjuvants or considering nonpharmacological pain management strategies may help decrease long-term utilization of opioids and development of tolerance in high risk patients.

Additionally, OAT, with methadone or buprenorphine, is crucial in the treatment of opioid use disorder, highlighted by the designation of methadone as an essential medication for its role in decreasing opioid-related overdoses [27,28]. The COVID-19 pandemic threatens the feasibility and safety of continued access to OAT, due to the possible closure of select treatment programs in regions where COVID-19 has already exhibited rapid community spread. To address this issue, multiple governmental mandates have been instituted to facilitate continued access to OAT. For example, Substance Abuse and Mental Health Services Administration issued a nationwide governance on 16 March 2020 allowing 28 day supplies of methadone for stable patients in an OTP and a 14 day supply for patients who are less stable [29], which was previously reserved for patients who have undergone therapy for at least 1 year. Substance Abuse and Mental Health Services Administration also issued recommendations regarding measures that should be taken to provide ‘doorstep delivery’ or surrogate pick-up for quarantined patients due to COVID-19 [30]. Regarding buprenorphine, fewer regulatory modifications have been instituted since the COVID-19 pandemic given the already established access via outpatient pharmacies. However, it is crucial to note that buprenorphine provider supply did not meet the clinical demand prior to COVID-19 [31] and the process of the X-waiver system to register and train new providers may impose a limit on increasing access to buprenorphine [32]. The above need not apply only to established patients who are already enrolled in OAT. Federal regulations during COVID-19 now permit utilizing telemedicine prior to initiating buprenorphine treatment for new patients, without an initial in-person encounter [33].

Overall, pain management providers can consider prescribing lower-risk opioids, such as tramadol, methadone or buprenorphine, when clinically appropriate, for select patients in an attempt to further mitigate risks of both COVID-19 exposure and opioid misuse.

## Conclusion

The COVID-19 pandemic has significantly altered the routine management of chronic pain with opioid therapy, prompting pain management physicians to adapt to this unique clinical environment. Based on our experience in the US epicenter of the COVID-19 pandemic, we highlight some considerations for providers when initiating or continuing opioid therapy during this unique time, as well as strategies to ensure that patients continue to receive quality care and monitoring, while attempting to decrease continued spread of COVID-19. It is crucial to weigh the risks and benefits of in-person encounters and to employ telemedicine and remote monitoring techniques whenever possible. However, for patients at greater risk who require in-person encounters, clinics must make arrangements so that social distancing is prioritized to decrease exposure risk for patients and healthcare staff. As we continue to adapt to the challenges at hand due to COVID-19, new technologies and innovations revolutionizing the practice of modern medicine may also advance techniques in the management of chronic opioid therapy and monitoring**.**
